# Association between polypharmacy and clinical outcomes in children with medical complexity: a retrospective cohort study

**DOI:** 10.3389/fped.2026.1826264

**Published:** 2026-07-09

**Authors:** Shuangzhu Shao, Kafen Hu, Jinlong Zhou

**Affiliations:** 1 Department of Pediatrics, The People’s Hospital of Danyang, Affiliated Danyang Hospital of Nantong University, Danyang City, Zhenjiang City, Jiangsu Province, China; 2 Department of Pharmacy, Zhangzhou Affiliated Hospital of Fujian Medical University, Zhangzhou City, Fujian Province, China; 3 Department of Pharmacy, Jiangsu Province Hospital of Chinese Medicine, Nanjing City, Jiangsu Province, China

**Keywords:** adverse drug events, children with medical complexity, healthcare utilization, medication safety, polypharmacy

## Abstract

**Importance:**

Children with medical complexity (CMC) frequently receive multiple medications, yet the impact of polypharmacy on clinical outcomes in this population remains inadequately characterized.

**Objective:**

To determine the association between polypharmacy and adverse drug events (ADEs), medication appropriateness, adherence, and healthcare utilization in CMC.

**Design, setting, and participants:**

Retrospective cohort study of 1,486 CMC aged 0–18 years at The People's Hospital of Danyang, Affiliated Danyang Hospital of Nantong University between January 2019 and June 2021, with minimum 12-month follow-up.

**Exposures:**

Polypharmacy was defined as concurrent use of 5 or more chronic medications for 90 or more consecutive days. PRN medications were excluded from the primary analysis.

**Main outcomes and measures:**

Primary outcomes were ADEs (Naranjo algorithm), potentially inappropriate medication (PIM) use (KIDs List), and medication adherence (proportion of days covered, PDC). Secondary outcomes included emergency department visits, hospital admissions, ICU admissions, hospital days, and 30-day readmissions.

**Results:**

Of 1,486 CMC, 628 (42.3%) had polypharmacy. Polypharmacy was associated with significantly higher ADE rates (5.5 vs 1.5 per patient-year; adjusted rate ratio 2.70; 95% CI 2.30–3.20), PIM use (53.8% vs 17.7%), ED visits (5.5 vs 2.3 per year), unplanned admissions (3.5 vs 1.3 per year), ICU admissions (43.3% vs 20.9%), and lower medication adherence (mean PDC 63.5% vs 80.2%) (all *P* < 0.001). A dose-response relationship was observed (*P* for trend < 0.001).

**Conclusions and relevance:**

Polypharmacy in CMC was strongly associated with increased ADEs, PIM use, reduced adherence, and elevated healthcare utilization. These findings warrant prospective studies of medication optimization in this population.

## Key points

**Question:** Is polypharmacy associated with adverse clinical outcomes, medication safety issues, and increased healthcare utilization in children with medical complexity?
**Findings:** In this cohort study of 1,486 CMC, those with polypharmacy (5 or more chronic medications for 90 or more days) had significantly higher rates of adverse drug events (aRR 2.70; 95% CI 2.30–3.20), PIM use (53.8% vs 17.7%), ED visits (5.5 vs 2.3 per year), unplanned admissions (3.5 vs 1.3 per year), and ICU admissions (43.3% vs 20.9%), with clear dose-response relationships across polypharmacy severity levels.
**Meaning:** These results highlight the need for prospective studies evaluating systematic medication optimization through multidisciplinary review, including deprescribing protocols when appropriate and enhanced medication reconciliation processes in this high-risk population.

## Introduction

Children with medical complexity (CMC) represent a small but clinically and economically significant subset of the pediatric population, characterized by multiple chronic conditions requiring ongoing subspecialty care, substantial functional limitations, and frequent dependence on medical technology for survival and quality of life 1–3. While comprising only an estimated 0.5% to 1% of all children in the United States, this vulnerable population accounts for a disproportionate 30% to 40% of total pediatric healthcare expenditures and experiences markedly elevated rates of healthcare utilization including emergency department visits, hospital admissions, and intensive care unit stays 4–6. The care complexity inherent to this population creates unique challenges for healthcare systems, families, and clinicians alike.

Polypharmacy, most commonly defined as the concurrent use of five or more medications, has emerged as a growing clinical and public health concern in adult populations and is increasingly recognized as an important issue in pediatric care settings 7–9. Unlike adults, where polypharmacy thresholds are relatively well-established, pediatric polypharmacy thresholds remain inconsistently applied due to the heterogeneity of chronic conditions, inter-individual variability in disease severity, and complex age-dependent pharmacokinetic and pharmacodynamic considerations 10–12. CMC are particularly vulnerable to polypharmacy exposure due to their multiple comorbidities, frequent involvement of multiple subspecialty providers, and inherently complex care needs 13–15.

Previous studies have documented the substantial prevalence of polypharmacy in CMC populations but have not comprehensively examined its multifaceted association with clinical outcomes and healthcare utilization patterns 16–18. Polypharmacy in children poses unique risks including heightened susceptibility to adverse drug events due to developing organ systems, increased risk of clinically significant drug-drug interactions (DDIs), elevated rates of medication errors due to complex regimens and weight-based dosing, and reduced medication adherence related to regimen complexity and caregiver burden 19–21.

Understanding the association between polypharmacy and clinical outcomes in CMC is essential for developing evidence-based interventions, optimizing medication regimens through systematic review and deprescribing protocols, and improving care quality. We hypothesized that CMC with polypharmacy would demonstrate significantly higher rates of adverse drug events, PIM use, reduced medication adherence, and increased healthcare utilization compared with CMC without polypharmacy, with dose-response relationships across increasing levels of polypharmacy severity. Given the retrospective observational design, this study aimed to identify associations that may inform future prospective research.

## Methods

### Study design and setting

We conducted a single-institution, retrospective cohort study in accordance with the Strengthening the Reporting of Observational Studies in Epidemiology (STROBE) guidelines 22. The study was conducted at The People's Hospital of Danyang, Affiliated Danyang Hospital of Nantong University, using data extracted from the hospital's electronic medical record system. The study was approved by the Ethics Committee of The People's Hospital of Danyang, Affiliated Danyang Hospital of Nantong University. Written informed consent was obtained from the participants or their legal guardians, as appropriate.

### Study population and eligibility criteria

We identified CMC receiving ongoing care between January 1, 2019 and June 30, 2021, with follow-up through June 30, 2022, ensuring a minimum of 12 months of follow-up for all patients. CMC were identified using the validated Pediatric Medical Complexity Algorithm (PMCA) 23, incorporating complex chronic conditions (CCC) as defined by Feudtner et al. (version 2) 3. Inclusion criteria required: (1) at least one CCC as defined by the CCC classification system version 2; (2) ongoing subspecialty care from two or more different pediatric subspecialty clinic types (at least one visit to each within the 12 months preceding enrollment); (3) either technology dependence (gastrostomy tubes, tracheostomy, home ventilation, or central venous catheters) or significant functional limitations; and (4) minimum 12 months continuous follow-up within the health system. The subspecialty care requirement applied only to care documented within our health system, as ascertainment of external subspecialty visits was not feasible. Exclusion criteria included incomplete records, transfer of care, and less than 90 days of medication data.

### Exposure definition and classification

The primary exposure was polypharmacy, defined *a priori* as concurrent use of 5 or more chronic medications for 90 or more consecutive days during the study period 24, 25. Chronic medications were those prescribed for ongoing management with regular scheduled administration. As-needed (PRN) medications (e.g., analgesics, rescue bronchodilators, laxatives) were excluded from the primary polypharmacy count because PRN use is inherently intermittent and difficult to quantify from electronic health record data alone; they were evaluated in sensitivity analyses. Vaccines, over-the-counter supplements without established pharmacological action (defined as supplements available without a prescription for which systemic pharmacological effects have not been demonstrated in peer-reviewed literature), and topical medications with minimal systemic absorption were also excluded. Patients whose medication count varied over time were classified according to their highest concurrent chronic medication count sustained for 90 or more consecutive days. For subgroup analyses, patients were categorized as: no polypharmacy (0–4 medications), standard polypharmacy (5–9), high polypharmacy (10–14), and extreme polypharmacy (15 or more).

### Data collection and variables

Patient data were collected by trained research assistants through standardized medical record review with dual independent data entry and discrepancy resolution. Demographic variables included age and sex. Socioeconomic variables included insurance type (Private, Medicaid, Dual Medicaid-Private, or Uninsured), household income (census tract estimates), and caregiver education level. Medical complexity variables included primary diagnosis category (neurological, cardiac, respiratory, gastrointestinal, metabolic, immunologic, or multisystem disorders) assigned through CCC classification; technology dependence (gastrostomy tube, tracheostomy, home ventilation, central venous catheter); Functional Status Scale (FSS; scores 6–30, higher = worse function) 26; and the number of unique subspecialty clinic types 23. Medication complexity was assessed using the Medication Regimen Complexity Index (MRCI) 27.

Medication data extracted from the electronic health record included medication name, dose, route, frequency, indication, start date, and discontinuation date. Medications were categorized according to the hospital formulary and medication classification system used at The People's Hospital of Danyang, Affiliated Danyang Hospital of Nantong University. Potential DDIs were assessed using the Micromedex Drug Interaction Checker (IBM Watson Health); clinically significant DDIs were those categorized as contraindicated, major, or moderate severity. PIMs were identified using the Key Potentially Inappropriate Drugs in Pediatrics (KIDs) List 28, 29, developed specifically for pediatric populations. Strong anticholinergics were defined as medications with an Anticholinergic Drug Scale (ADS) score of 2 or 3 30.

### Outcome definitions

Primary outcomes were: (1) ADEs per patient-year, identified through chart review for medication-related adverse events, ED visits or hospitalizations attributed to medications, and laboratory abnormalities consistent with drug toxicity. ADE causality was assessed using the Naranjo Adverse Drug Reaction Probability Scale, with events scoring 5 or more (probable or definite) classified as ADEs; two investigators independently reviewed events, with discrepancies resolved by discussion or adjudication by a third clinical pharmacist; inter-rater reliability was assessed using Cohen kappa. (2) PIM use defined by the KIDs List 28; (3) Medication adherence assessed as proportion of days covered (PDC) over 12 months, with prescription gaps exceeding 7 days considered non-adherent and PDC 80% or higher defined as good adherence 31, 32.

Secondary outcomes included ED visits, unplanned hospital admissions (excluding scheduled procedures), ICU admissions, cumulative hospital days, and 30-day readmission rates. Composite medication-related morbidity was defined as the occurrence of any of: ADE requiring medical intervention, clinically significant DDI (contraindicated, major, or moderate per Micromedex), or medication error identified through the institutional safety reporting system.

### Statistical analysis

Characteristics were compared between polypharmacy and non-polypharmacy groups using chi-square or Fisher exact tests for categorical variables, and independent t-tests or Wilcoxon rank-sum tests for continuous variables based on normality assessed by the Shapiro–Wilk test. FSS scores were non-normally distributed (all Shapiro–Wilk *P* < 0.001); therefore, medians with interquartile ranges are reported. *P* values are two-tailed with italicized P, and *P* < 0.05 considered statistically significant.

ADE rates were analyzed using negative binomial regression to account for overdispersion. Binary outcomes were analyzed using logistic regression. Adherence was analyzed both as continuous PDC (linear regression) and binary (logistic regression). Multivariable models adjusted for age, sex, insurance type, primary diagnosis category, and number of subspecialty clinic types. Technology dependence and FSS were considered potential mediators and excluded from primary models to avoid overadjustment bias; models additionally adjusted for these variables are presented as sensitivity analyses. Collinearity was assessed using variance inflation factors (VIF < 5 acceptable). Model fit was assessed using R-squared, deviance, and AUC as appropriate.

The primary endpoint was ADE rate per patient-year. The Benjamini-Hochberg procedure controlled the false discovery rate at 5% across secondary outcomes. Subgroup analyses were pre-specified by age group (0–2, 3–5, 6–12, 13–18 years), presence of technology dependence, and primary diagnosis category (neurological vs non-neurological). The neurological subgroup was pre-specified based on the hypothesis that medications with narrow therapeutic indices and enzyme-inducing/inhibiting properties (e.g., antiepileptic drugs) may differentially modify the polypharmacy-outcome association.

Sensitivity analyses included: varying polypharmacy thresholds (6 or more, 7 or more, 8 or more medications); restricting to complete follow-up patients; including PRN medications in the medication count; alternative PIM definitions; and 1:1 propensity score matching (nearest-neighbor without replacement, caliper 0.2 SD). Missing data (less than 20% for all variables) were handled using multiple imputation (20 datasets, fully conditional specification). Analyses were performed using SAS 9.4 and R 4.2.0 33, with a comprehensive statistical analysis plan finalized prior to data analysis.

## Results

### Patient characteristics

**Figure 1:**
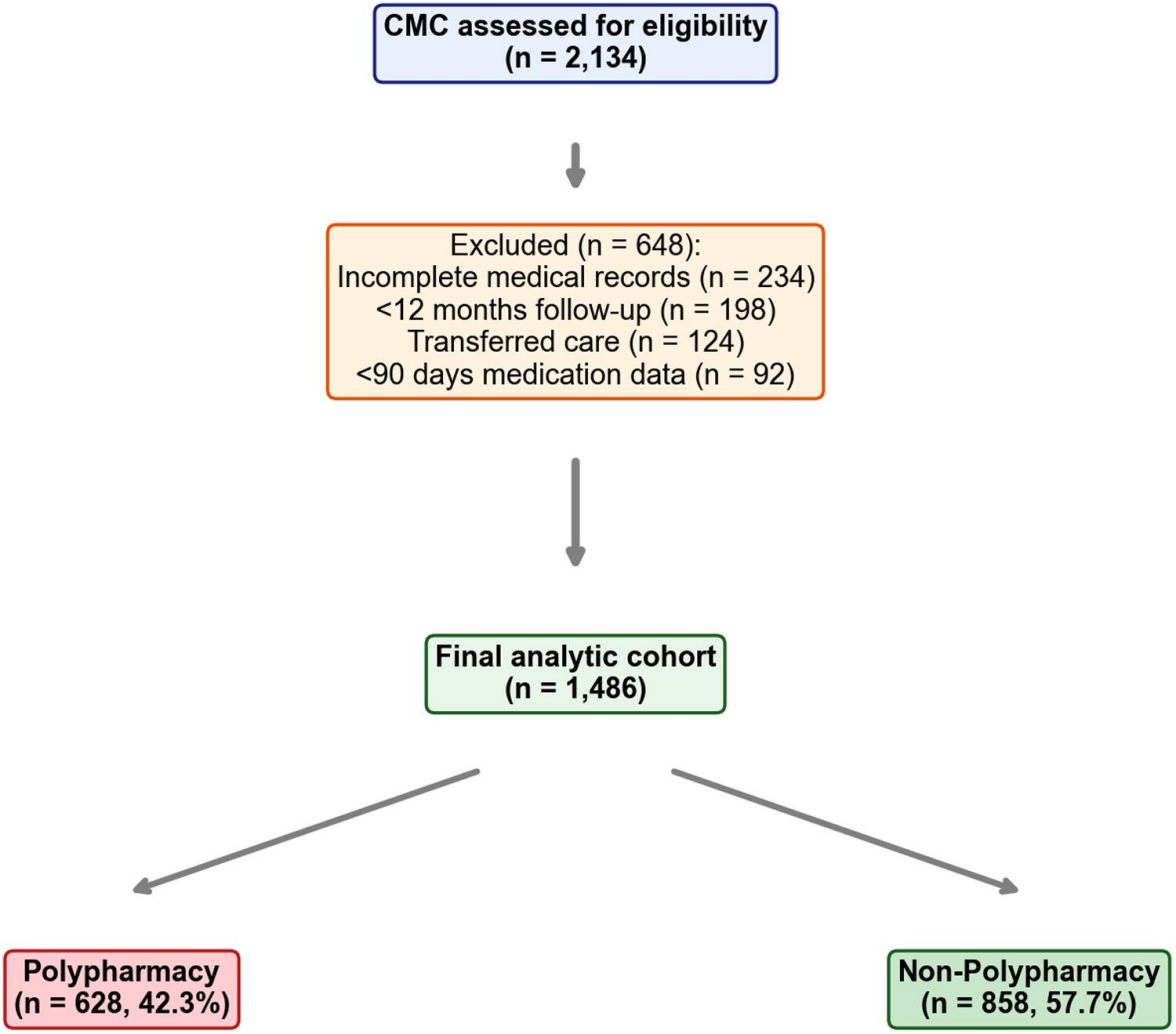
STROBE flow diagram. Flow diagram illustrating the patient selection process. A total of 2,134 CMC were screened, of whom 648 were excluded (incomplete records *n* = 234; less than 12 months follow-up *n* = 198; transferred care *n* = 124; less than 90 days medication data *n* = 92). The final analytic cohort comprised 1,486 patients (polypharmacy *n* = 628; non-polypharmacy *n* = 858).

A total of 1,486 CMC met inclusion criteria (STROBE flow diagram, Figure 1); 628 (42.3%) were classified in the polypharmacy group and 858 (57.7%) in the non-polypharmacy group (Table 1). Patients in the polypharmacy group were older [mean [SD] age 9.4 [4.5] vs 6.5 [3.9] years; *P* < 0.001] and had a higher proportion of males [366 of 628 [58.3%] vs 454 of 858 [52.9%]; *P* = 0.045].

**Table 1:** Patient characteristics by polypharmacy Status.

Variable	Non-Polypharmacy (*n* = 858)	Polypharmacy (*n* = 628)
**Demographics**		
Age, mean (SD), y	6.5 (3.9)	9.4 (4.5)
Male, No. (%)	454 (52.9)	366 (58.3)
**Insurance type, No. (%)**		
Medicaid	489 (57.0)	422 (67.2)
Private	327 (38.1)	178 (28.3)
Dual Medicaid-Private	27 (3.1)	15 (2.4)
Uninsured	15 (1.7)	13 (2.1)
Household income, mean (SD), $	59,045 (24,120)	54,019 (20,905)
Caregiver college education, No. (%)	349 (40.7)	230 (36.6)
**Primary diagnosis category, No. (%)**		
Neurological	259 (30.2)	417 (66.4)
Cardiac	104 (12.1)	28 (4.5)
Gastrointestinal	117 (13.6)	31 (4.9)
Respiratory	113 (13.2)	38 (6.1)
Metabolic	79 (9.2)	28 (4.5)
Immunologic	41 (4.8)	22 (3.5)
Multisystem	145 (16.9)	64 (10.2)
Technology dependence, No. (%)	416 (48.5)	510 (81.2)
Gastrostomy tube	147 (17.1)	288 (45.9)
Tracheostomy	50 (5.8)	116 (18.5)
Home ventilation	34 (4.0)	91 (14.5)
Central venous catheter	37 (4.3)	81 (12.9)
FSS, median (IQR)	12 (9−16)	18 (13–23)
Subspecialty clinic types, mean (SD)	3.2 (1.3)	5.8 (2.2)
MRCI, mean (SD)	9.8 (5.0)	28.7 (11.1)

Socioeconomic indicators revealed important disparities. The polypharmacy group had higher rates of Medicaid coverage [422 of 628 [67.2%] vs 489 of 858 [57.0%]] and lower rates of private insurance [178 of 628 [28.3%] vs 327 of 858 [38.1%]; *P* < 0.001 for overall insurance distribution]. Dual Medicaid-Private coverage [15 of 628 [2.4%] vs 27 of 858 [3.1%]] and uninsured status [13 of 628 [2.1%] vs 15 of 858 [1.7%]] were uncommon in both groups. Household income was lower in the polypharmacy group [mean [SD] $54,019 [$20,905] vs $59,045 [$24,120]; *P* < 0.001]. Caregiver college education did not differ significantly between groups [230 of 628 [36.6%] vs 349 of 858 [40.7%]; *P* = 0.13].

Medical complexity indicators demonstrated substantial differences (Table 1). The polypharmacy group had markedly higher rates of neurological impairment as the primary diagnosis [417 of 628 [66.4%] vs 259 of 858 [30.2%]; *P* < 0.001], consistent with the high medication burden often required for seizure management, spasticity, and associated conditions. Primary diagnosis categories shifted substantially from cardiopulmonary and gastrointestinal conditions in the non-polypharmacy group toward neurological disorders in the polypharmacy group (cardiac 12.1% vs 4.5%; gastrointestinal 13.6% vs 4.9%; respiratory 13.2% vs 6.1%). Technology dependence was more prevalent in the polypharmacy group [510 of 628 [81.2%] vs 416 of 858 [48.5%]; *P* < 0.001], with significant differences in gastrostomy tubes [288 of 628 [45.9%] vs 147 of 858 [17.1%]; *P* < 0.001], tracheostomy [116 of 628 [18.5%] vs 50 of 858 [5.8%]; *P* < 0.001], home ventilation [91 of 628 [14.5%] vs 34 of 858 [4.0%]; *P* < 0.001], and central venous catheters [81 of 628 [12.9%] vs 37 of 858 [4.3%]; *P* < 0.001]. FSS scores were non-normally distributed (Shapiro–Wilk *P* < 0.001 for both groups) and indicated greater impairment in the polypharmacy group [median (IQR) 18 13–23 vs 12 9–16; *P* < 0.001]. The number of subspecialty clinic types was higher for polypharmacy patients [mean [SD] 5.8 [2.2] vs 3.2 [1.3]; *P* < 0.001].

### Medication characteristics

**Figure 2:**
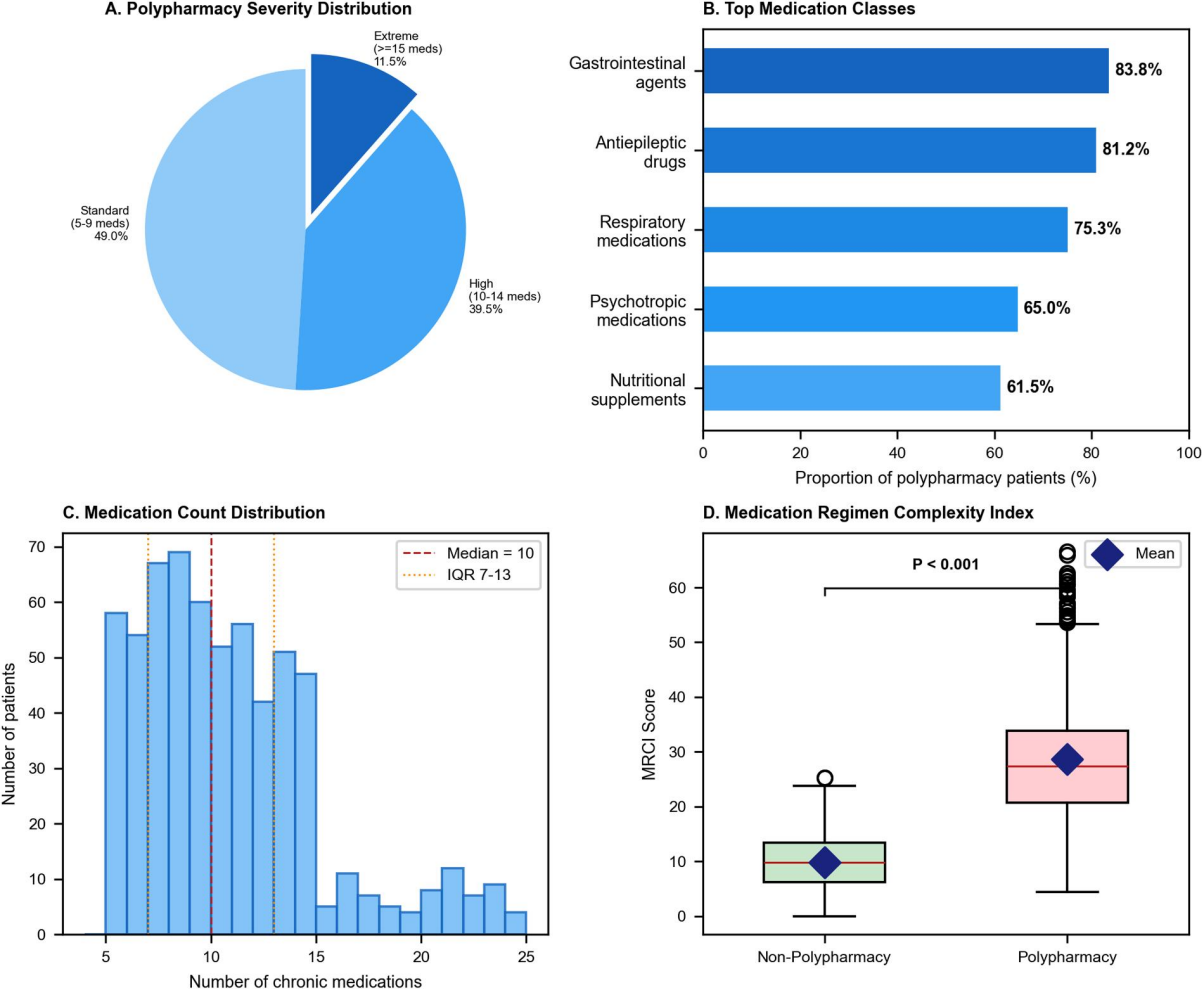
Medication characteristics in the polypharmacy cohort (*n* = 628). **(A)** Polypharmacy severity: standard (5−9 medications) 49.0%, high (10−14) 39.5%, extreme (15 or more) 11.5%. **(B)** Top five medication classes: gastrointestinal agents (83.8%), antiepileptic drugs (81.2%), respiratory medications (75.3%), psychotropic medications (65.0%), nutritional supplements (61.5%). **(C)** Medication count distribution (median 10, IQR 7−13, range 5−24). **(D)** MRCI score comparison between non-polypharmacy and polypharmacy groups (9.8 +/- 5.0 vs 28.7 +/- 11.1; *P* < 0.001). Error bars indicate SD.

Among the polypharmacy group, the median number of concurrent chronic medications was 10 (IQR 7–13; range 5–24), with 308 patients (49.0%) meeting criteria for standard polypharmacy (5–9 medications), 248 (39.5%) for high polypharmacy (10–14), and 72 (11.5%) for extreme polypharmacy (15 or more). The most commonly prescribed medication classes were gastrointestinal agents [526 of 628 (83.8%)], antiepileptic drugs [510 of 628 (81.2%)], respiratory medications [473 of 628 (75.3%)], psychotropic medications [408 of 628 (65.0%)], and nutritional supplements with pharmacological effects [386 of 628 (61.5%)]. The MRCI score was substantially higher in the polypharmacy group [mean [SD] 28.7 [11.1] vs 9.8 [5.0]; *P* < 0.001], reflecting both the number and complexity of medication regimens (Figures 2A–D). The most common DDI class combinations involved antiepileptic drugs with gastrointestinal agents (68.0% of polypharmacy patients), gastrointestinal agents with respiratory medications (63.5%), and antiepileptic drugs with respiratory medications (61.6%).

### Clinical outcomes

ADEs occurred with significantly greater frequency in the polypharmacy group [mean [SD] 5.5 [6.2] vs 1.5 [1.6] events per patient-year; *P* < 0.001], representing a more than threefold increase (Table 2). This difference remained significant after multivariable adjustment (aRR 2.70; 95% CI 2.30–3.20; *P* < 0.001). ADE causality assessment using the Naranjo algorithm yielded a total of 3,457 events in the polypharmacy group vs 1,259 in the non-polypharmacy group (mean Naranjo score 6.3 vs 6.4; Cohen kappa = 0.86; 95% CI 0.84–0.88). Polypharmacy patients had substantially more moderate-to-severe ADEs requiring medical intervention (494 vs 356 patient-episodes) and serious ADEs requiring hospitalization (182 vs 33 patient-episodes).

**Table 2:** Clinical outcomes and healthcare utilization by polypharmacy Status.

Outcome	Non-Polypharmacy (*n* = 858)	Polypharmacy (*n* = 628)	*P* value
**Adverse Drug Events**			
ADEs per patient-year, mean (SD)	1.5 (1.6)	5.5 (6.2)	<0.001
Total ADE events (Naranjo >= 5)	1,259	3,457	<0.001
Moderate-severe ADE episodes	356	494	<0.001
ADE requiring hospitalization, episodes	33	182	<0.001
**Medication Appropriateness**			
PIM use (KIDs List), No. (%)	152 (17.7)	338 (53.8)	<0.001
Clinically significant DDIs, No. (%)	97 (11.3)	230 (36.6)	<0.001
**Medication Adherence**			
PDC, mean (SD), %	80.2 (8.5)	63.5 (11.9)	<0.001
Good adherence (PDC >= 80%), No. (%)	447 (52.1)	46 (7.3)	<0.001
**Healthcare Utilization**			
ED visits per year, mean (SD)	2.3 (2.3)	5.5 (5.3)	<0.001
Unplanned admissions per year, mean (SD)	1.3 (1.5)	3.5 (3.9)	<0.001
ICU admission, No. (%)	179 (20.9)	272 (43.3)	<0.001
Total hospital days per year, mean (SD)	9.8 (12.4)	27.4 (33.2)	<0.001
30-day readmission, No. (%)	182 (21.2)	222 (35.4)	<0.001
Composite morbidity, No. (%)	629 (73.3)	578 (92.0)	<0.001

PIM use, as identified by the KIDs List, was markedly elevated in the polypharmacy group [338 of 628 [53.8%] vs 152 of 858 [17.7%]; *P* < 0.001; aOR 3.25; 95% CI 2.60–4.07]. Clinically significant DDIs were more common in the polypharmacy group [230 of 628 [36.6%] vs 97 of 858 [11.3%]; *P* < 0.001].

Medication adherence was substantially lower in the polypharmacy group [mean [SD] PDC 63.5% [11.9%] vs 80.2% [8.5%]; *P* < 0.001; adjusted mean difference −14.8%; 95% CI −17.2% to −12.4%]. Good adherence (PDC 80% or higher) was achieved by only 46 of 628 polypharmacy patients (7.3%) vs 447 of 858 non-polypharmacy patients (52.1%); *P* < 0.001; aOR 0.42; 95% CI 0.34–0.52), representing a 44.8 percentage point absolute difference.

### Healthcare utilization

Healthcare utilization was elevated across all measured metrics in the polypharmacy group (Table 2). ED visits occurred more frequently [mean [SD] 5.5 [5.3] vs 2.3 [2.3] per year; *P* < 0.001; aRR 1.95; 95% CI 1.70–2.24]. Unplanned hospital admissions were more common [mean [SD] 3.5 [3.9] vs 1.3 [1.5] per year; *P* < 0.001; aRR 2.12; 95% CI 1.79–2.52]. ICU admissions occurred in 272 of 628 polypharmacy patients (43.3%) vs 179 of 858 non-polypharmacy patients (20.9%; *P* < 0.001; aOR 2.18; 95% CI 1.72–2.76). Total hospital days were more than doubled [mean [SD] 27.4 [33.2] vs 9.8 [12.4] days; *P* < 0.001]. Thirty-day readmission rates were markedly elevated [222 of 628 [35.4%] vs 182 of 858 [21.2%]; *P* < 0.001]. The composite medication-related morbidity outcome occurred in 578 of 628 (92.0%) polypharmacy patients vs 629 of 858 (73.3%) non-polypharmacy patients (*P* < 0.001; aOR 2.86; 95% CI 2.29–3.57).

### Dose-Response and subgroup analyses

**Figure 3:**
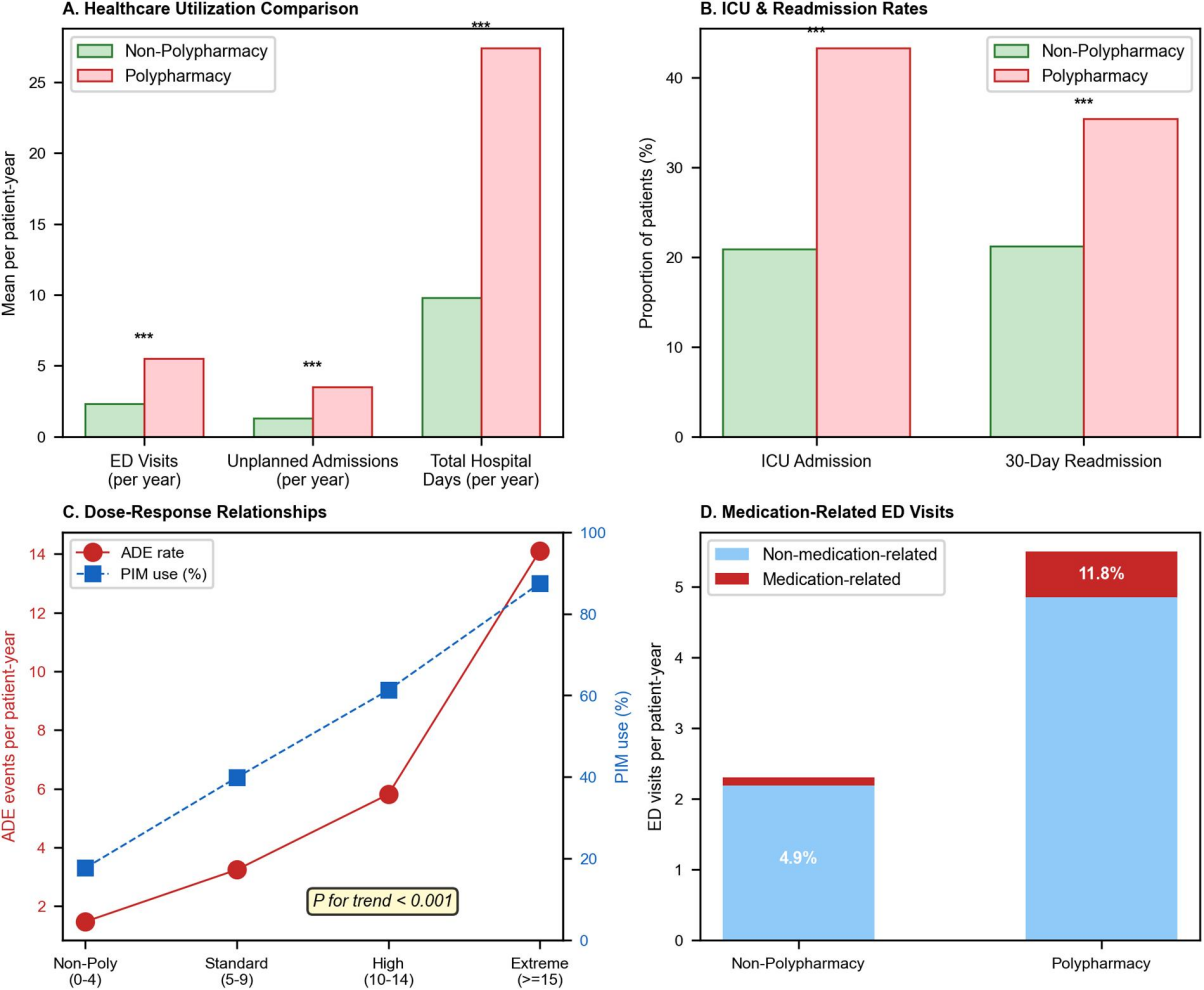
Healthcare utilization by polypharmacy Status. **(A)** Polypharmacy group showed more than 2-fold increases in ED visits, hospital admissions, and hospital days (*P* < 0.001). **(B)** ICU admissions (43.3% vs 20.9%) and 30-day readmissions (35.4% vs 21.2%) were significantly elevated in polypharmacy patients (*P* < 0.001). **(C)** Dose-response trends across polypharmacy severity categories (*P* for trend < 0.001). **(D)** Medication-related ED visits as a proportion of total ED utilization: 11.8% vs 4.9% (2.4-fold increase in the polypharmacy group).

Dose-response analyses revealed progressively worse outcomes across polypharmacy severity categories (Figures 3A–D). ADE rates increased from 1.47 (non-polypharmacy) to 3.24 (standard), 5.81 (high), and 14.11 (extreme) events per patient-year (*P* for trend < 0.001). PIM use rose from 17.7% to 39.9%, 61.3%, and 87.5% respectively. PDC declined from 80.2% to 69.8%, 60.2%, and 47.9%. ICU admission rates rose from 20.9% to 34.1%, 46.8%, and 70.8%. These trends remained significant after Benjamini-Hochberg correction.

Pre-specified subgroup analyses showed consistent associations across age groups (*P* for interaction = 0.34) and by technology dependence status (*P* for interaction = 0.21). The exploratory neurological subgroup showed an ADE aRR of 3.54 vs 3.49 in the non-neurological subgroup (*P* for interaction = 0.04), warranting cautious interpretation.

Sensitivity analyses varying the polypharmacy threshold to 6 or more, 7 or more, and 8 or more medications yielded consistent results (unadjusted ADE rate ratios of 3.87, 3.84, and 3.91, respectively). Including PRN medications produced an unadjusted ADE rate ratio of 3.22. Propensity score matching demonstrated consistent findings across all outcomes. Results from all sensitivity analyses are presented in the Supplementary Material.

## Discussion

To our knowledge, this study represents one of the largest and most comprehensive cohort analyses examining the multifaceted association between polypharmacy and clinical outcomes in CMC. We found that polypharmacy was strongly and independently associated with substantially increased ADEs (aRR 2.70), elevated PIM use (53.8% vs 17.7%), markedly reduced medication adherence (mean PDC 63.5% vs 80.2%), and elevated healthcare utilization across multiple metrics including ED visits (aRR 1.95), unplanned admissions (aRR 2.12), and ICU admissions (aOR 2.18). Our findings extend previous research documenting polypharmacy prevalence in CMC 7–9 by demonstrating clear, measurable clinical consequences across a broader array of outcomes than previously reported 14, 15.

The more than threefold increase in ADEs among children with polypharmacy represents a major patient safety concern. Our application of the Naranjo algorithm for causality assessment strengthens ADE ascertainment beyond physician-documented attribution alone. These results align with both adult literature demonstrating increased ADE risk with polypharmacy 19 and pediatric studies highlighting vulnerability due to developing organ systems, age-dependent pharmacokinetics, and medication administration challenges 10, 11. The increased ADE risk is likely attributable to multiple overlapping mechanisms: predictable pharmacological effects account for a substantial proportion; DDIs represent another important contributor (36.6% prevalence of clinically significant DDIs in the polypharmacy group vs 11.3% in non-polypharmacy); medication errors in complex weight-based regimens may further compound these risks. Specific strategies warrant consideration: systematic medication reconciliation at each clinical encounter, therapeutic drug monitoring for narrow-therapeutic-index medications (particularly antiepileptic drugs), substitution with agents having lower interaction potential, and integration of clinical decision support tools that prospectively identify DDIs before medication initiation 16, 18.

The finding of substantially reduced medication adherence in the polypharmacy group is clinically important, as poor adherence likely contributes to inadequate disease control and potentially unnecessary medication intensification. The 44.8 percentage point absolute difference in achieving good adherence (PDC 80% or higher) between groups represents a clinically meaningful gap. The MRCI score was substantially higher in the polypharmacy group (28.7 vs 9.8), suggesting that regimen complexity directly contributes to caregiver burden and administration challenges 20, 21. Potential solutions include regimen simplification through consolidated dosing times and extended-release formulations, combination products to reduce pill burden, enhanced caregiver education (teach-back methods, written medication action plans), and telemonitoring support including pharmacist-led telephone follow-up and mobile health technology for medication reminders.

The elevation in healthcare utilization has important implications for both patient care and health system resource allocation 14, 15. The near-doubling of ED visits, more than doubling of unplanned admissions, and nearly threefold increase in hospital days translate into considerable healthcare costs, family disruption, and potential iatrogenic complications from hospitalizations 16–18. The observed association between Medicaid insurance and polypharmacy prevalence warrants consideration: Medicaid-insured CMC may face barriers including fewer participating subspecialists, restricted formularies, and care coordination challenges 15; conversely, greater disease severity may simultaneously drive both Medicaid eligibility (through disability-based pathways) and higher medication burden.

The clear dose-response relationship across polypharmacy severity categories, with ADE rates rising from 1.47 to 14.11 events per patient-year across categories, strengthens the credibility of the observed association and is consistent with a biological gradient. However, given the retrospective observational design, this study can only establish associations rather than causation. Confounding by indication remains a fundamental limitation: children with more severe underlying conditions may require more medications and simultaneously be at higher risk for the adverse outcomes studied. The finding that 53.8% of polypharmacy patients had at least one PIM highlights substantial opportunities for medication optimization. The identification of specific PIM classes through the validated KIDs List provides clinicians with concrete, actionable targets for medication review. Chronic proton pump inhibitor use without clear ongoing indication has been associated with increased risks of community-acquired pneumonia and Clostridioides difficile infection, particularly concerning for CMC with underlying respiratory or gastrointestinal conditions. Chronic benzodiazepine use in children without seizure disorders raises concerns regarding cognitive effects, physical dependence, and potential respiratory depression, especially in patients with concurrent respiratory compromise. Medications with strong anticholinergic properties (ADS score 2 or higher) contribute to cumulative anticholinergic burden, which has been associated with cognitive impairment, constipation, and urinary retention in pediatric populations.

Clinical pharmacists play a critical role within multidisciplinary medication management programs, with specialized expertise to lead medication reviews, conduct comprehensive medication reconciliation across transitions of care, identify PIMs using validated tools such as the KIDs List, provide caregiver education on medication administration and adherence strategies, and ensure ongoing monitoring for ADEs and DDIs. Previous studies in adult populations have demonstrated that pharmacist-led medication reviews can reduce polypharmacy, PIM use, and ADE-related hospitalizations 16, 18; extending these models to pediatric CMC populations through prospective studies represents a critical research priority. Several deprescribing frameworks developed for adult populations 34 could be adapted for pediatric use with incorporation of pediatric-specific considerations including weight-based dosing, developmental pharmacokinetics, and family-centered decision-making.

Although this is a single-center study, the biological relationship between polypharmacy and adverse outcomes is likely consistent across populations and healthcare settings. The dose-response relationship, consistency across extensive sensitivity analyses, and biological plausibility of the observed associations suggest that the relationship may be generalizable, though the magnitude of effect may differ across settings with different case-mix, prescribing practices, and healthcare delivery structures 35.

## Limitations

This study has several important limitations. First, it was a single-institution retrospective cohort study at a large academic children's hospital, which may limit external generalizability to community hospitals, different geographic regions, or international contexts.

Second, the observational design prevents definitive causal inference. Despite extensive adjustment, unmeasured confounding, particularly by disease severity, remains a fundamental limitation. Although the dose-response relationship strengthens the credibility of the association, it does not establish causation.

Third, regarding temporal ambiguity: polypharmacy was defined as a fixed baseline characteristic with prospective outcome observation over 12 months. However, reverse causation remains a concern; a child experiencing an ADE leading to hospitalization could receive additional medications during admission, potentially influencing exposure classification. We classified patients based on their first qualifying polypharmacy period and began outcome ascertainment after the 90-day exposure window, but this cannot fully eliminate reverse causation.

Fourth, outcome and exposure definitions have conceptual overlaps. ED visits documented as medication-related contribute to both ADE rates and healthcare utilization metrics. PIM use is mechanistically linked to ADE risk, potentially introducing endogeneity; we addressed this by analyzing these outcomes in separate regression models.

Fifth, medication adherence was measured using prescription refill data (PDC). In the CMC population, where many medications are administered via gastrostomy tubes or during hospitalizations, prescription refills reflect pharmacy pickup rather than actual administration. Ideal adherence assessment would require caregiver logs, electronic monitoring, or direct clinical inquiry, which were not feasible in this retrospective study design.

Sixth, healthcare utilization was captured only within The People's Hospital of Danyang, Affiliated Danyang Hospital of Nantong University, which may have underestimated healthcare utilization occurring at other institutions.

Seventh, the clinical acuity leading to ICU admission may reflect underlying disease severity rather than medication-related factors in some cases. Finally, the KIDs List represents expert consensus rather than empirical outcome data for all included medications; ongoing validation of pediatric PIM criteria against clinical outcomes is needed.

## Conclusions

In this comprehensive retrospective cohort study of 1,486 children with medical complexity, polypharmacy was strongly and independently associated with increased adverse drug events (aRR 2.70; 95% CI 2.30–3.20), potentially inappropriate medication use (53.8% vs 17.7%), substantially reduced medication adherence (mean PDC 63.5% vs 80.2%; aOR 0.42), and dramatically elevated healthcare utilization across multiple important metrics including ED visits (aRR 1.95), unplanned hospital admissions (aRR 2.12), and ICU admissions (aOR 2.18). These associations demonstrated clear dose-response relationships across polypharmacy severity categories (*P* for trend < 0.001), persisted after extensive multivariable adjustment, and were consistent across multiple sensitivity analyses. The findings warrant prospective studies to test whether systematic medication review and optimization protocols led by clinical pharmacists, including appropriate deprescribing strategies and enhanced medication reconciliation processes, improve clinical outcomes and reduce healthcare burden in this high-risk pediatric population.

## Data Availability

The datasets analyzed during the current study are not publicly available due to patient privacy and ethical restrictions, but de-identified data may be available from the corresponding author upon reasonable request and with approval from the relevant ethics committee. The names of the repository/repositories and accession number(s) can be found in the article/Supplementary Material.
