# Supplementary material for: Association between polypharmacy and clinical outcomes in children with medical complexity: a retrospective cohort study
Source: Front Pediatr. 2026 Jul 9;14:1826264. doi: 10.3389/fped.2026.1826264 (PMC13391845; doi:10.3389/fped.2026.1826264)
Supplement: Supplementary file 1 [file Supplementaryfile1.docx]

Supplementary Material

Association Between Polypharmacy and Clinical Outcomes in Children With Medical Complexity: A Retrospective Cohort Study

Shuangzhu Shao, Kafen Hu, Jinlong Zhou

# Supplementary Table S1

Dose-response relationships across polypharmacy severity categories for all clinical outcomes.

| **Polypharmacy Category** | **n** | **ADE Rate (per pt-yr)** | **PIM Use (%)** | **PDC (%)** | **ED Visits (per yr)** | **Admissions (per yr)** | **ICU Admission (%)** | **Composite Morbidity (%)** |
| --- | --- | --- | --- | --- | --- | --- | --- | --- |
| Non-polypharmacy 0-4 | 858 | 1.47 | 17.7 | 80.2 | 2.33 | 1.32 | 20.9 | 73.3 |
| Standard 5-9 | 308 | 3.24 | 39.9 | 69.8 | 4.11 | 2.47 | 34.1 | 86.7 |
| High 10-14 | 248 | 5.81 | 61.3 | 60.2 | 5.51 | 3.56 | 46.8 | 96.4 |
| Extreme >=15 | 72 | 14.11 | 87.5 | 47.9 | 10.97 | 7.93 | 70.8 | 100.0 |

All P for trend < 0.001 (Benjamini-Hochberg corrected). Non-polypharmacy (0-4 medications), Standard (5-9), High (10-14), Extreme (>=15).

# Supplementary Table S2

Sensitivity analyses varying polypharmacy threshold definitions and including PRN medications.

| **Definition** | **Exposed (n)** | **Unexposed (n)** | **ADE Rate Ratio** | **ED Visit Rate Ratio** | **Admission Rate Ratio** | **PIM Risk Difference (%)** | **PDC Mean Difference (%)** |
| --- | --- | --- | --- | --- | --- | --- | --- |
| >=5 chronic meds | 628 | 858 | 3.74 | 2.34 | 2.67 | 36.1 | 16.7 |
| >=6 chronic meds | 570 | 916 | 3.87 | 2.39 | 2.77 | 38.2 | 17.4 |
| >=7 chronic meds | 516 | 970 | 3.84 | 2.32 | 2.82 | 38.5 | 18.0 |
| >=8 chronic meds | 449 | 1037 | 3.91 | 2.31 | 2.96 | 41.5 | 18.8 |
| >=5 total meds including PRN | 857 | 629 | 3.22 | 2.15 | 2.41 | 27.2 | 13.3 |

All unadjusted rate ratios. The >=5 total meds including PRN analysis includes both chronic and as-needed medications in the count.

# Supplementary Table S3

Full multivariable regression model results for all primary and secondary outcomes.

| **Outcome** | **Model** | **Effect Measure** | **Estimate** | **95% CI Lower** | **95% CI Upper** | **P Value** |
| --- | --- | --- | --- | --- | --- | --- |
| Primary ADE rate | Negative binomial | Adjusted rate ratio | 2.70 | 2.30 | 3.20 | <0.001 |
| PIM use | Logistic regression | Adjusted odds ratio | 3.25 | 2.60 | 4.07 | <0.001 |
| PDC adherence percent | Linear regression | Adjusted mean difference | -14.80 | -17.20 | -12.40 | <0.001 |
| Good adherence PDC >=80% | Logistic regression | Adjusted odds ratio | 0.42 | 0.34 | 0.52 | <0.001 |
| ED visits | Negative binomial | Adjusted rate ratio | 1.95 | 1.70 | 2.24 | <0.001 |
| Unplanned admissions | Negative binomial | Adjusted rate ratio | 2.12 | 1.79 | 2.52 | <0.001 |
| ICU admission | Logistic regression | Adjusted odds ratio | 2.18 | 1.72 | 2.76 | <0.001 |
| Composite medication-related morbidity | Logistic regression | Adjusted odds ratio | 2.86 | 2.29 | 3.57 | <0.001 |

All models adjusted for age, sex, race/ethnicity, insurance type, primary diagnosis category, and number of subspecialty clinic types. Negative binomial models used for count outcomes (ADE rate, ED visits, unplanned admissions); logistic regression for binary outcomes; linear regression for continuous PDC.

# Supplementary Table S4

Adverse drug event causality assessment using the Naranjo Adverse Drug Reaction Probability Scale.

| **Naranjo Category** | **Score Range** | **Total Events** | **Polypharmacy** | **Non-Polypharmacy** |
| --- | --- | --- | --- | --- |
| Definite (>=9) | 9-13 | 259 | 189 | 70 |
| Probable (5-8) | 5-8 | 4457 | 3268 | 1189 |
| Total (>=5) | 5-13 | 4716 | 3457 | 1259 |

Cohen kappa = 0.86 (95% CI 0.84-0.88). ADE causality assessed independently by two investigators with discrepancies resolved by discussion or adjudication by a third clinical pharmacist.

# Supplementary Table S5

Most common potentially inappropriate medication (PIM) classes identified by the KIDs List in the polypharmacy group (n=628).

| **AHFS Drug Class** | **Patients (n)** | **Proportion (%)** |
| --- | --- | --- |
| Gastrointestinal agents | 139 | 22.1 |
| Antiepileptic drugs | 136 | 21.7 |
| Psychotropic medications | 107 | 17.0 |
| Respiratory medications | 37 | 5.9 |
| Vitamins/minerals pharmacologic | 34 | 5.4 |
| Cardiovascular agents | 32 | 5.1 |
| Anticholinergics | 20 | 3.2 |
| Antibiotics/anti-infectives | 16 | 2.5 |
| Analgesics | 13 | 2.1 |
| Endocrine/metabolic agents | 13 | 2.1 |

# Supplementary Table S6

Pre-specified and exploratory subgroup analyses for ADE rate by polypharmacy status.

| **Subgroup** | **Polypharmacy (n)** | **Non-Polypharmacy (n)** | **ADE Rate (poly)** | **ADE Rate (non-poly)** | **Unadjusted RR** |
| --- | --- | --- | --- | --- | --- |
| Age 0-2 years | 34 | 125 | 5.53 | 1.30 | 4.27 |
| Age 3-5 years | 61 | 148 | 5.23 | 1.37 | 3.81 |
| Age 6-12 years | 302 | 384 | 5.61 | 1.46 | 3.84 |
| Age 13-18 years | 137 | 49 | 5.70 | 1.92 | 2.97 |
| With technology dependence | 510 | 416 | 5.72 | 1.57 | 3.64 |
| Without technology dependence | 118 | 442 | 4.57 | 1.37 | 3.34 |
| Primary diagnosis: Neurological | 417 | 259 | 5.83 | 1.65 | 3.54 |
| Primary diagnosis: Non-neurological | 211 | 599 | 4.85 | 1.39 | 3.49 |

Age subgroup P for interaction = 0.34. Technology dependence P for interaction = 0.21. Neurological vs. non-neurological P for interaction = 0.04 (exploratory).

# Supplementary Table S7

Sensitivity analysis: additional multivariable adjustment for Functional Status Scale and technology dependence (potential mediators).

| **Outcome** | **Effect Measure** | **Primary Model Estimate (95% CI)** | **FSS+Tech Adjusted Estimate (95% CI)** |
| --- | --- | --- | --- |
| Primary ADE rate | aRR | 2.70 (2.30-3.20) | 2.12 (1.78-2.53) |
| PIM use | aOR | 3.25 (2.60-4.07) | 2.47 (1.95-3.13) |
| ED visits | aRR | 1.95 (1.70-2.24) | 1.62 (1.40-1.88) |
| Unplanned admissions | aRR | 2.12 (1.79-2.52) | 1.74 (1.45-2.09) |
| ICU admission | aOR | 2.18 (1.72-2.76) | 1.68 (1.31-2.16) |
| Composite morbidity | aOR | 2.86 (2.29-3.57) | 1.98 (1.56-2.52) |

Attenuation of effect estimates after adjusting for potential mediators (FSS and technology dependence) is consistent with partial mediation. Primary models excluding these covariates represent the total effect of polypharmacy on outcomes.
